# The Impact of the Concussion Crisis on Safeguarding in Sport

**DOI:** 10.3389/fspor.2021.589341

**Published:** 2021-02-25

**Authors:** Dominic Malcolm

**Affiliations:** School of Sport, Exercise and Health Sciences, Loughborough University, Loughborough, United Kingdom

**Keywords:** brain injury, chronic traumatic encephalopathy, concussion, safeguarding, regulation, medicine

## Introduction

For ~30 years, sociologists have explored the distinct ways in which athletes experience injury (Young, 2019). This work has explained the apparent high incidence of injury in relation to subcultural factors such as the dominance of masculinity (McGannon et al., 2013) and the specific organizational dynamics of sport (Nixon, 1992). Walk (1997, p. 24) perceptively noted that the implication of these analyses was that “medicine is practiced differently, more competently, and/or more ethically in non-sports contexts,” a hypothesis that has largely been borne out by subsequent empirical analyses (Malcolm, 2017). Indicatively, a study of English professional football concluded that “many clubs fail to meet the requirements of health and safety legislation” (Murphy and Waddington, 2011, p. 239). A high incidence of injury allied to limited or substandard healthcare runs contrary to the guiding principles of safeguarding in sport.

In the last decade, rising public health concerns about brain injuries in sport—both concussion and chronic traumatic encephalopathy (CTE)—have reinvigorated this field. Specifically, as coroners decreed that the neurodegenerative illnesses of former athletes were a form “industrial disease,” questions were asked about the sport's subcultural practices and a range of harm reduction measures were initiated. The previous routine dismissal of concussions as short-lived and relatively trivial events has been replaced by claims that there is now a concussion epidemic or crisis (Malcolm, 2020). Public support for sports injury safeguarding measures is perhaps stronger now than at any time in recent history.

This opinion piece explores the impact of this concussion crisis on injury prevention and safeguarding in sport. It outlines the significant changes that have been made in recent years and the problematic or potentially limiting aspects of these changes. The discussion identifies three far-reaching changes required to promote further safeguarding and de-institutionalize physical harm to sport participants.

## How Awareness of Concussion Has Made Sport Safer

Concerns about brain injuries in sport stimulated political inquiries which have subsequently forced sports organizations to reconsider their duties to protect participants at all playing levels. Most notably, in a 2009 Congressional hearing the National Football League (NFL) were accused of neglect in both protecting current players from injury and managing the cases of retired players exhibiting cognitive decline. The response of the NFL was to repeatedly cast doubt and uncertainty over the reliability of scientific evidence and the causal connection between *playing* the game and player harm (Goldberg, 2013). Congress viewed the NFL as having fallen “short of community and government expectations” and failing “to regulate its sport for and in the best interests of the players and the public” (Greenhow and East, 2015, p. 75, 76). In 2015, a class action lawsuit concluded when the NFL agreed to establish a $1bn player compensation fund. These events were clearly and vividly depicted in the influential film and book *League of Denial* (Fainaru-Wada and Fainaru, 2013), and the publicity surrounding the NFL's experiences alerted sports governing bodies around the world to their duties of care, candor (or openness), and diligence in actively searching for relevant information about the physical harms to players (Anderson, 2016).

In response to their duty of care, governing bodies of most (if not all) contact sports have integrated specific clauses about the management of concussion into their rules and regulations. Many sports have instigated rule changes which seek to limit the extent or type of contact between players/playing equipment (McGannon et al., 2013; Cassilo and Sanderson, 2018). Sports teams are now constrained in their management of concussion through requirements to undertake sideline evaluations, restrictions on the return to play of diagnosed players, and the use of concussion spotters or non-affiliated doctors to assess head injuries. They are further enabled through adaptations to substitution regulations for players with suspected concussions. Many sports have also instigated their own concussion awareness or education programmes. Player education programmes are mandatory in youth sport across the USA following the adoption of the Lystedt Law, while similar provisions are likely to be introduced across Canada following the adoption of Rowan's Law in Ontario.

In response to their duty of diligence, governing bodies for many contact sports have funded research programmes. Studies have focused on quantifying the incidence of injury (West et al., 2020), assessing harm mitigation developments (e.g., improved helmet design, the impact of new laws and training programmes), and (occasionally) charting the longer-term neurocognitive decline of retired athletes (Hume et al., 2017; Mackay et al., 2019). Such research is inherently controversial. While failure to fund scientific research can lead to allegations of neglect (e.g., in the case of the National Hockey League), commissioning research can be interpreted as undermining the essential neutrality of scientific investigation and thus unduly influencing the evidence base. Undertaking due diligence can also evoke criticisms about governing bodies' duty of candor (Carlisle, 2018). In 2015, World Rugby clashed with their academic investigator over the interpretation of research findings (https://www.nzherald.co.nz/sport/news/article.cfm?c_id=4&objectid=11658681) and in 2017 was forced to retract some of the claims made in publicity materials about the relative safety of the sport (Piggin and Pollock, 2017).

The consequence of these combined changes is that the protection of concussed athletes is greater than at any point in history. Overall, however, the concussion crisis has meant that governing bodies have a heightened awareness of the injuries participants routinely experience, more proactively investigate health risks, and have enacted harm reduction changes.

## The Limits of Change

Despite these developments, three features of the concussion crisis effectively restrict the degree of protection offered by these harm mitigation policies.

First, all existing concussion protocols are premised on the assumption that injuries should be managed as discrete rather than cumulative events. Specifically, while both the immediate withdrawal of players suspected of being concussed and graduated return to play (GRTP) protocols represent important safeguarding measures, there are no regulations which govern players who experience multiple concussions. The empirical evidence is contested but, at the very least, data indicating that symptoms may become more frequent and severe, and potentially also lead to longer-term neurocognitive decline, suggest the “first do no harm” medical ethical principle is *in*consistently applied (McNamee et al., 2015).

Second, regulations consistently treat children more conservatively than adults, e.g., through longer “normal recovery” times and elongated GRTP protocols (McCrory et al., 2017). Again the empirical evidence is inconsistent and paternalistic concerns largely account for the restrictions placed on youth sport involvement. It is not clear why mandatory concussion education (e.g., under the Lystedt Law) should be implemented for children and not adults. Paternalistic protection of the child must not obscure the need to address the harms experienced by adults. A more logical response to studies which associate the development of neurocognitive conditions with exposure to sport (Mackay et al., 2019) is to restrict the maximum duration of participants' playing careers.

Third, policies have been introduced with seemingly naïve expectations of compliance. Qualitative research demonstrates the challenges healthcare professionals experience in pursuing medical best practice in sports contexts (Malcolm, 2018) and indicates that stricter regulation may be counterproductive (Malcolm, 2009). Cusimano et al. (2017) argued that concussion protocols were followed in just 37% of cases during the 2014 FIFA World Cup. Others have pointed to a lack of monitoring and effective sanction to properly enforce concussion regulations in both Australia (Partridge, 2014) and the USA (Mrazik et al., 2015).

## Discussion: The Future of Concussion and Safeguarding

The two sides of the concussion crisis debate remain polarized between those who insist that greater restrictions on individual liberty are justified by the need to protect participants (especially children) from harm, and those who believe the value of sports participation (especially physical and mental health benefits) outweigh the potential risks (Quarrie et al., 2017). Consequently, the regulation of concussion in sport remains a “wicked problem”; complex, difficult to define and continuously evolving (Greenhow and East, 2015). These tensions are likely to fuel continuous incremental safeguarding adaptations, but more radical reforms will require three main reconsiderations.

1. Reconfiguring the regulatory environment. The guiding ideologies of public health and sports medicine are, respectively, the prevention of harm/promotion of safety and enabling participation and performance (Safai, 2003). Consequently, these two groups view the standards of evidence and burdens of proof required for concussion-related changes from different ends of a spectrum. For as long as sport governing bodies retain their current levels of autonomy over concussion regulation, the public health lobby will remain frustrated. However, the historic consensus that sports should operate a significant level of self-governance has been eroded in recent years, particularly in relation to financial management, doping, and child protection. This creates a precedent for further changes to athlete healthcare. Momentum may now be sufficient to initiate collaborative state-sport approaches—akin perhaps to the dual funding of the World Anti-Doping Agency—and hence bring these often opposing medical fields together in the regulation of sport.
2. Reinforcing the ethical norms of medical management. The context, clients and co-workers experienced in sport create distinct pressures on healthcare workers (Malcolm, 2017). Bucking societal trends, there is a clear case for greater medical autonomy in sport as a way of addressing the problems Walk (1997) initially identified. The use of “neutral” medical staff to aid sideline concussion assessments is a step in this direction, but these measures are limited to diagnosis rather than ongoing management and rehabilitation from injury. Securer contractual arrangements, more rigorous appointment procedures, and greater oversight from the medical profession will not only help raise the standards of healthcare for concussion, but for all types of injury in sport (Waddington et al., 2019).
3. Invoking *comprehensive* cultural change. Sustained and enduring change requires a cultural shift. All stakeholders—owners, coaches, athletes, medical staff, the media, parents, educators—have a role to play in questioning sport cultural norms around the tolerance of pain and injury (Frey, 1991; Hughes and Coakley, 1991), acceptance of harm, and celebration of risk-taking (Liston et al., 2018; Matthews, 2020). While the concussion crisis has been effective in raising social awareness of these issues, a major unintended consequence of extended regulation has been to position concussion as a unique form of injury. Consequently, this precautionary stance may be unnecessarily restricted to concussion injuries. Further safeguarding in sport requires these underlying precautionary principles to be transferable to other injury risks. Paradoxically, making concussion *un*exceptional is necessary for the relatively high injury rates to be more effectively challenged.

## Author Contributions

The author confirms being the sole contributor of this work and has approved it for publication.

## Conflict of Interest

The author declares that the research was conducted in the absence of any commercial or financial relationships that could be construed as a potential conflict of interest.
